# Pictorial review of cardiac sarcomas and their mimickers

**DOI:** 10.1186/1470-7330-14-S1-P36

**Published:** 2014-10-09

**Authors:** Luba Frank, Jadranka Stojanovska

**Affiliations:** 1 University of Michigan, Ann Arbor, MI, USA

## Purpose

This review will present a spectrum of cardiac sarcomas and mimicking conditions in order to provide tools for differentiation.

## Background

Cardiac sarcoma remains one of the most aggressive primary cardiac tumours, which can arise from different cardiac chambers and demonstrate various features, depending on differentiation and histologic composition. In many cases the diagnosis is difficult, and consequences of incorrect diagnosis are too harmful.

We will present different types of cardiac sarcomas and their imaging features by MRI and CT in selected cases.

We will also show and discuss features of sarcomas like conditions, presenting diagnostic challenge, such as pulmonary artery thrombus mimicking angiosarcoma, fibrosing mediastinitis, infiltrative primary cardiac lymphoma, benign tumours, and thrombi.

## Summary

This review aims to present imaging characteristics of cardiac sarcomas, their most important mimickers and benign differential diagnoses in order to avoid potential pitfalls, guide to achieve proper diagnosis, leading to timely and appropriate treatment.

